# Whipple’s disease scleral nodules: a novel presentation in 2 consecutive patients

**DOI:** 10.1186/s12886-020-01695-4

**Published:** 2020-10-16

**Authors:** Waleed K. Alsarhani, Muhannad I. Alkhalifah, Hind M. Alkatan, Afaf L. Alsolami, Azza M. Y. Maktabi, Adel H. Alsuhaibani

**Affiliations:** 1 Ophthalmology Department, College of Medicine, King Saud University, Riyadh, Saudi Arabia; 2 Pathology Department, College of Medicine, King Saud University Medical city, King Saud University, Riyadh, Saudi Arabia; 3 Pathology and Laboratory Medicine, King Khaled Eye Specialist Hospital, Riyadh, Saudi Arabia

**Keywords:** Whipple’s disease, Scleritis, Scleral nodule, Tropheryma whipplei

## Abstract

**Background:**

Whipple’s disease (WD) is a rare, chronic, infection caused by gram-positive filamentous aerobic actinobacterium Tropheryma whipplei occurs classically in the gastrointestinal tract and shows histopathologically foamy macrophages with typical numerous PAS-positive, non-acid fast particles. Ocular WD in the form of uveitis may occur in the absence of systemic disease but has not been reported to present with scleral manifestation. We describe for the first time to the best of our knowledge 2 cases of scleral nodules with typical histopathological morphology of WD and without systemic involvement.

**Case presentation:**

The first was a 53-year old diabetic male farmer who presented with 2 nontender right eye scleral nodules for 3 months, had a negative systemic workup, and surgical excision showed Periodic acid Schiff (PAS)-positive eosinophilic structures inside macrophages. Grocott’s methenamine silver (GMS) stain and acid-fast bacilli (AFB) stain of the tissue itself were negative. The second case was a 60-year old male who presented with an asymptomatic superior scleral nodule for 4 months, which showed similar appearance and negative GMS and AFB stains.

**Conclusion:**

WD should be included in the differential diagnosis of scleral nodules even in the absence of systemic symptoms. Surgical excision without systemic treatment resulted in successful outcome without recurrence.

## Background

Whipple’s disease (WD) is a rare, chronic, multi-organ systemic infection caused by gram-positive or gram-intermediate aerobic filamentous actinobacterium Tropheryma whipplei [1]. The presentation can be classic, in which gastrointestinal (GI) symptoms and weight loss is the hallmark, or isolated, which has been seen in 17% of patients. Ocular involvement, typically in the form of uveitis, has been described in 11% of the patients [1, 2]. Isolated scleral involvement has never been reported in the literature to the best of our knowledge. Herein, we report two cases of three painless scleral nodules with histopathologic evidence of WD.

## Case presentation

### Case 1

**Fig. 1:**
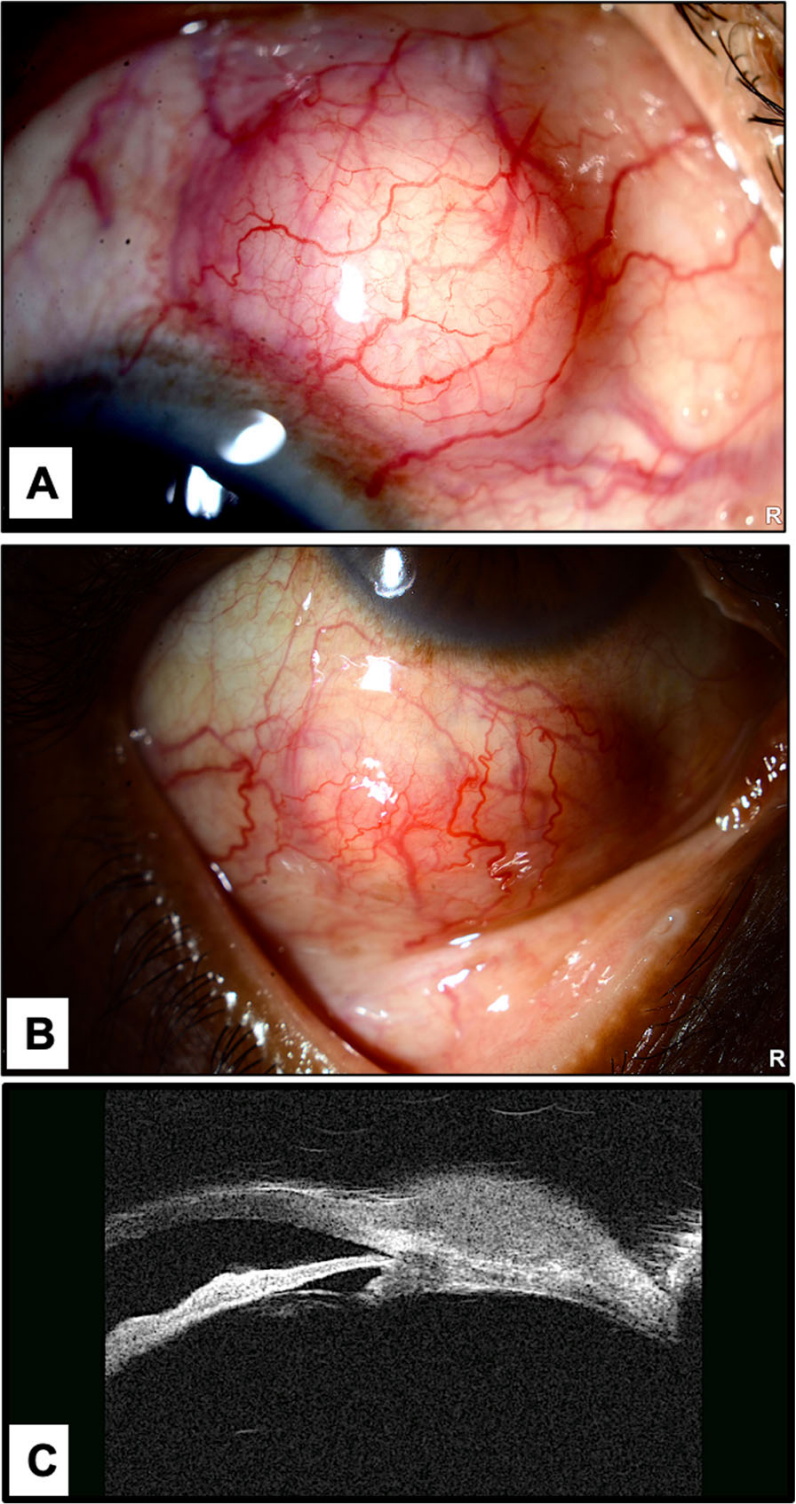
**a** and **b** The clinical appearance of the 2 round scleral nodules located superiorly and inferiorly surrounded by feeder vessels in case 1 (Phenylephrine drops were not used). **c** Ultrasound Biomicroscopy of case 1 showing homogenous hypo-reflective subconjunctival lesion overlying an area of scleral thinning. **d** Intra-operative photo demonstrating purulent material within the nodule

**Fig. 2:**
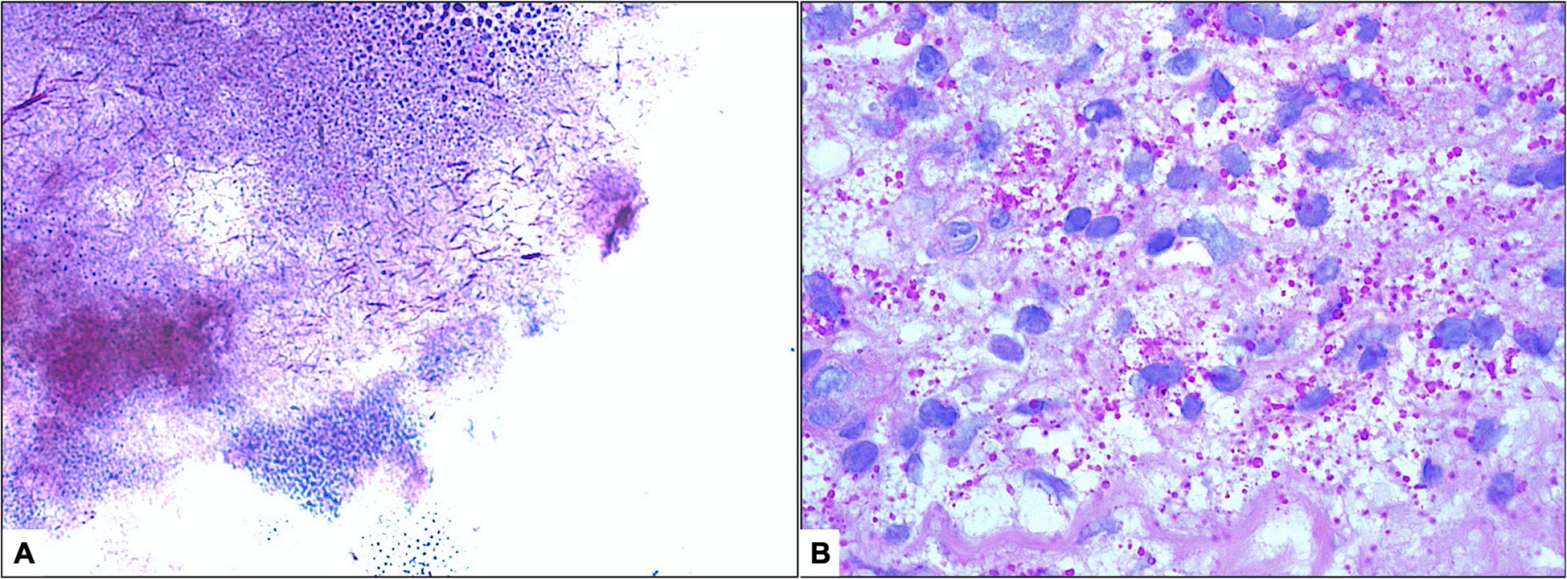
**a** Gram-positive filamentous organisms within the purulent exudate (Original magnification × 1000-oil, Gram stain). **b** Histopathological photo of the tissue excised in case 1 clearly demonstrating the large amounts of Diastase-resistant intracytoplasmic organisms within foamy macrophages (Original magnification × 1000-oil, Periodic acid Schiff with diastase)

A 53-year old Saudi diabetic male farmer presented to our ophthalmology clinic with 2 subconjunctival scleral nodules in his right eye for 3 months. The nodules were stable in size, and the patient did not complain of any ocular symptoms until his recent presentation. His past medical history was significant for diabetic foot, for which he underwent above knee amputation and was receiving antidiabetic medications. He denied history of trauma, weight loss, joint pain, diarrhea, abdominal pain and any neurological symptoms. Visual acuity was 20/25 in both eyes, and the extraocular motility was full without nystagmus or signs of oculomasticatory myorhythmia. There was two nontender scleral nodules located superiorly and inferiorly in the right eye (Fig. 1a and b). The nodules were surrounded by dilated vessels while the remaining conjunctiva was quiet. The anterior chamber was deep and quiet with mild iris neovascularization in the right eye and dot blot hemorrhages and microaneurysms in all quadrants on posterior pole exam. Examination of the left eye was unremarkable except for severe non-proliferative diabetic retinopathy. Ultrasound bio-microscopy (UBM) showed homogenous subconjunctival lesions (Fig. 1c). Systemic workup including tuberculin skin test (TST), chest X-ray was, rheumatoid factor, antineutrophil cytoplasmic antibodies, and angiotensin converting enzyme was negative. The patient was booked for excisional biopsy with conjunctival advancement by an experienced ophthalmologist. Intraoperatively the nodule was filled with pus (Fig. 1d) with underlying scleral melting, but no areas of scleral perforation were noted to necessitate the use of scleral patch or amniotic membrane transplant. A sample obtained from the nodule was sent for microbiological assessment which revealed numerous gram-positive filamentous organisms (Fig. 2a). The histopathology of the excised scleral tissue showed fibrosis and lymphoplasmacytic cells infiltration with foamy macrophages (Fig. 2b). Periodic acid Schiff (PAS) stain with/and without diastase demonstrated numerous tiny eosinophilic structures inside the macrophages that represent Trophyrema whipplei. Grocott’s methenamine silver stain (GMS), Gram and acid-fast bacilli (AFB) stains were negative. Histopathology slides were reviewed by three different pathologists. After discussion with the internal medicine and infectious disease specialists and since the patient did not develop any systemic features, a decision not to start systemic antibiotics was taken. However, post-operative topical Ofloxacin 0.3% drops QID and Prednisolone acetate 1.0% drops QID with tapering dose over 1 month were used in the operated eye as standard topical medications following such a procedure. There was no recurrence over 6 months of follow up.

### Case 2

**Fig. 3:**
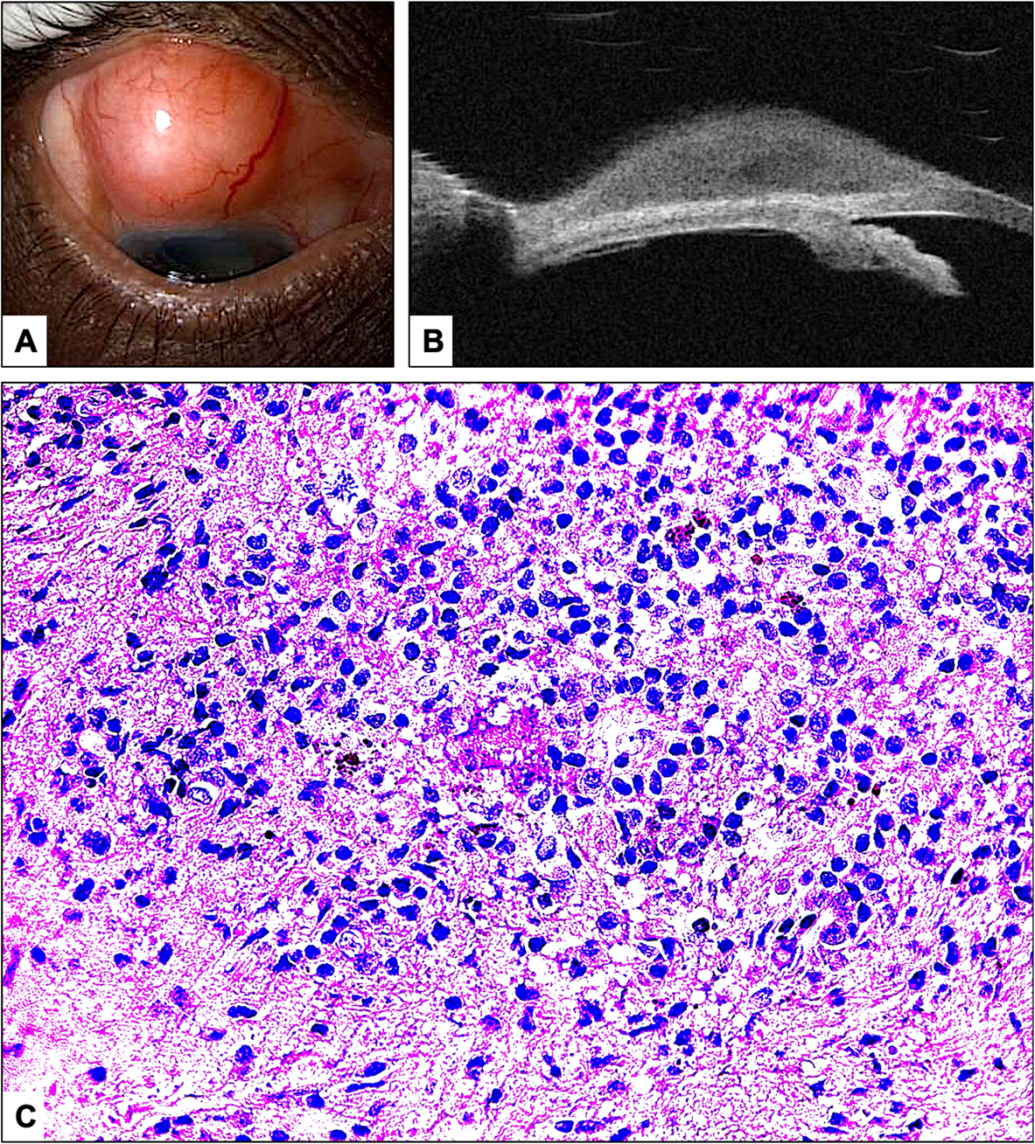
**a** The clinical photo of the superior round subconjunctival nodule in case 2. Note the quiet surrounding conjunctiva. **b** Ultrasound bio-microscopy similarly showing homogenous subconjunctival lesion. **c** Similar numerous tiny intracytoplasmic organisms within macrophages and adjacent chronic inflammatory cells in the scleral tissue of case 2 (Original magnification × 400, Periodic acid Schiff)

A 60-year old Saudi male presented with a superior scleral nodular swelling for 4 months with no change in size over time. Past medical history was unremarkable except for asthma. He denied history of trauma, weight loss, joint pain, diarrhea, abdominal pain and any neurological symptoms and his drug history was unremarkable. On physical examination, visual acuity measured 20/30 and 20/25 in in the right and left eyes, respectively. There was a nontender round subconjunctival nodule located superiorly measuring 13 × 7 mm in size (Fig. 3a). Dilated fundus examination was within normal limits in both eyes. Examination of the other eye was unremarkable. Extraocular motility was full without nystagmus or signs of oculomasticatory myorhythmia. UBM showed a hyporeflective homogenous subconjunctival nodule (Fig. 3b). Systemic workup including TST, chest X-ray was, rheumatoid factor, antineutrophil cytoplasmic antibodies, and angiotensin converting enzyme was negative. The patient was evaluated by an internist, who stated the patient did not have any systemic condition except asthma.

The patient was booked for incisional biopsy by his treating ophthalmologist. Histopathology results showed fibrotic scleral tissue with chronic inflammatory cells infiltration including lymphocytes, plasma cells, and focal foamy macrophages showing similar numerous tiny PAS- positive structures (Fig. 3c). GMS, Gram and AFB stains were also negative. Histopathology slides were reviewed by three different pathologists. The diagnosis was made after the patient’s discharge, and on follow up visits, the patient showed satisfactory results with the use of topical antibiotics and Prednisolone acetate drops with tapering dose in the operated eye similar to the first case. He was also monitored for any systemic features or recurrence. Eventually, there was no recurrence over 4 months of follow up. Systemic antibiotics were not started since the patient did not develop any systemic or other ocular signs, and the scleral nodules did not recur.

## Discussion and conclusion

Nodular scleral swelling is a heterogenous finding reported in a wide variety of ocular pathologies. Congenital choristoma and multiple ocular tumors (i.e Solitary fibrous tumor, xanthogranuloma) can present as painless scleral masses [3]. Autoimmune inflammatory conditions, particularly sarcoidosis, are frequently reported with subconjunctival granulomas and scleral nodules [4]. Infectious scleral nodules were reported as a manifestation of ocular tuberculosis [5]. The advances in ocular imaging modalities with the help of histopathologic evaluation and molecular testing were helpful to reach a diagnosis in such disorders [3]. We reached the diagnosis in our cases after exclusion of other causes of scleral nodules and further histopathological confirmation of WD.

Asymptomatic WD may occur in 1–38% particularly in sewage workers [6]. However, no previous reports looked at the seroprevalence of WD in Saudi Arabia. Eye involvement in WD might occur in a minority of patients. Uveitis is the most common presentation but it may also present as retinitis, optic neuritis and even as lenticular epithelial changes [1, 2, 7]. Nodular scleral involvement has never been reported with WD to the best of our knowledge. Despite the known inflammatory nature of the different known forms of ocular WD, both of the reported cases did not have any evidence of ocular surface inflammation around the nodules. Ocular WD might occur in the absence of systemic disease, and several cases of WD uveitis were reported in the absence of GI disease [8, 9].

The histopathologic evaluation of tissue biopsy with the support of immunohistochemistry and polymerase chain reaction (PCR) remains the standard diagnostic test for Tropheryma whipplei [1]. The hallmark of histologic sections in WD are foamy macrophages containing large amounts of PAS-positive, non-acid fast particles in the lamina propria of GI mucosa [1]. The differential diagnosis of PAS-positive material inside macrophages include *Mycobacterium avium* complex, and histoplasmosis [10]. In both cases, AFB and fungal stains were done to rule out other causes of PAS-positive inclusions [5]. In ocular WD, in the presence of clear histopathological evidence, PCR may not be needed and may be required in equivocal cases [11]. Long-term antimicrobial treatment is required for systemic WD, many agents are described for induction and maintaining remission [1]. Both of our patients did not have any systemic disease nor posterior uveitis necessitating systemic treatment.

In conclusion, WD should be included in the differential diagnosis of scleral nodules even in the absence of systemic symptoms. We believe that isolated involvement of the sclera can happen in a similar way to the previously reported uveal tissue WD, but we do not have explanation for the way the organism might gain access to this scleral tissue. The accessibility of the lesions in this external location for diagnostic biopsy should be utilized to confirm the diagnosis. Surgical excision without systemic treatment resulted in successful outcome without recurrence.

## Data Availability

Data sharing was not applicable to this article, as no datasets were generated or analysed during the current study.

## Abbreviations

*WD*: Whipple’s disease
*PAS*: Periodic acid Schiff.
*GMS*: Grocott’s methenamine silver
*AFB*: Acid fast bacilli
*PCR*: Polymerase chain reaction.
